# Changes of Metabolites in Acute Ischemic Stroke and Its Subtypes

**DOI:** 10.3389/fnins.2020.580929

**Published:** 2021-01-11

**Authors:** Xin Wang, Luyang Zhang, Wenxian Sun, Lu-lu Pei, Mengke Tian, Jing Liang, Xinjing Liu, Rui Zhang, Hui Fang, Jun Wu, Shilei Sun, Yuming Xu, Jian-Sheng Kang, Bo Song

**Affiliations:** ^1^Department of Neurology, The First Affiliated Hospital of Zhengzhou University, Zhengzhou, China; ^2^Henan Key Laboratory of Cerebrovascular Diseases, Zhengzhou, China

**Keywords:** ischemic stroke, metabolites, non-targeted metabolomics, TOAST, biomarkers

## Abstract

Existing techniques have many limitations in the diagnosis and classification of ischemic stroke (IS). Considering this, we used metabolomics to screen for potential biomarkers of IS and its subtypes and to explore the underlying related pathophysiological mechanisms. Serum samples from 99 patients with acute ischemic stroke (AIS) [the AIS subtypes included 49 patients with large artery atherosclerosis (LAA) and 50 patients with small artery occlusion (SAO)] and 50 matched healthy controls (HCs) were analyzed by non-targeted metabolomics based on liquid chromatography–mass spectrometry. A multivariate statistical analysis was performed to identify potential biomarkers. There were 18 significantly different metabolites, such as oleic acid, linoleic acid, arachidonic acid, L-glutamine, L-arginine, and L-proline, between patients with AIS and HCs. These different metabolites are closely related to many metabolic pathways, such as fatty acid metabolism and amino acid metabolism. There were also differences in metabolic profiling between the LAA and SAO groups. There were eight different metabolites, including L-pipecolic acid, 1-Methylhistidine, PE, LysoPE, and LysoPC, which affected glycerophospholipid metabolism, glycosylphosphatidylinositol-anchor biosynthesis, histidine metabolism, and lysine degradation. Our study effectively identified the metabolic profiles of IS and its subtypes. The different metabolites between LAA and SAO may be potential biomarkers in the context of clinical diagnosis. These results highlight the potential of metabolomics to reveal new pathways for IS subtypes and provide a new avenue to explore the pathophysiological mechanisms underlying IS and its subtypes.

## Introduction

Stroke is one of the main causes of human death and disability (Wang et al., 2014) and is associated with a high rate of disability and recurrence. According to population-based studies (Benjamin et al., 2017), ischemic stroke (IS) accounts for more than 80% of all strokes. According to its etiology and imaging, IS can be categorized into five subtypes (Adams et al., 1993; Chen et al., 2012), including large artery atherosclerosis (LAA), small artery occlusion (SAO), cardioembolism, stroke of other determined cause, and stroke of undetermined cause. The classification of IS can help with the early treatment and prevention of long-term recurrence in patients (Montaner et al., 2008). However, the diagnosis and classification of IS mainly rely on neuroimaging techniques, which are scarce, expensive, and time-consuming (Latchaw et al., 2009). Therefore, new biomarkers for the rapid and accurate prediction, diagnosis, and classification of IS might play a positive role in clarifying the pathophysiological mechanism of IS and promoting the secondary prevention and management of patients with IS.

It is difficult to release macromolecules from the brain into the blood due to the presence of the blood–brain barrier (Jickling and Sharp, 2015). Some conventional detection methods make it difficult to detect specific sensitive different metabolites in patients with IS. However, with the development of the emerging science of metabolomics, it may be possible to identify specific small molecular biomarkers in patients with IS and determine the underlying etiology. Metabolomics is an effective method to reveal biomolecules’ phenotypes. This method enables the identification of changes in small molecular metabolites in various diseases, which can greatly help in understanding and diagnosing diseases. Many studies using metabolomics have revealed the differences in metabolites between patients with acute ischemic stroke (AIS) and healthy controls (HCs) (Jung et al., 2011; Kimberly et al., 2013). To date, few studies have explored the differences in metabolites between the LAA and SAO subtypes of IS. In this study, non-targeted metabolites based on liquid chromatography–mass spectrometry (LC–MS) were used to study the different metabolites between patients with AIS and the HCs and between patients with the LAA and SAO subtypes of IS. The proposed method offers important advantages over traditional alternatives, ensuring that it is feasible to screen potential biomarkers and further explore the relevant underlying pathophysiological mechanisms.

## Materials and Methods

### Study Population

In this study, 99 patients with AIS within 7 days of onset were included in the AIS group, including 49 patients with LAA and 50 patients with SAO. Among the 99 AIS patients, the time from onset to blood withdrawal was within 24 h among 45 patients and within 72 h among 38 patients. The remaining 16 patients showed transient ischemic attack symptoms within 7 days, but the blood samples were only collected at around 72 h after the symptoms begin to persist. A total of 50 HCs with age, sex, and risk factors matched with the AIS group were recruited. All patients needed to meet the following conditions: (1) no history of stroke or coronary heart disease, (2) no history of malignant tumor or autoimmune disease, (3) blood samples can be obtained within 24 h of enrollment, and (4) head magnetic resonance imaging and angiography were completed during hospitalization. The patients in the AIS group, who were hospitalized in the Department of Neurology from October 2015 to December 2016, and the samples of the AIS group were acquired from the ischemic cerebrovascular disease database and blood database of The First Affiliated Hospital of Zhengzhou University. The details of the database and related articles have been published elsewhere (Song et al., 2013; Kelly et al., 2016; Wang et al., 2020). All patients with AIS were diagnosed according to the diagnostic criteria of the World Health Organization [WHO] (1989). The TOAST classification was evaluated back to back by two professional neurologists. Written informed consent was obtained from all participants or their representatives.

### Serum Sample Preparation

Blood samples of patients with AIS were collected within 24 h after admission; when collecting, it was ensured that the patients have fasted for at least 8 h. The serum was centrifuged and extracted within 1 h and refrigerated at −80°C. To separate metabolites with different polarities, the same sample underwent two different treatment methods. After melting in ice at 4°C for 30–60 min, 40 μl of serum was taken into a 1.5-ml centrifuge tube for a reversed-phase ultra-performance liquid chromatographic analysis, adding 300 μl methanol and 1 ml methyl tert-butyl ether to precipitate the protein for 15 s. The sample was then placed in a centrifuge at 12,000 rpm at a constant temperature of 4°C for 10 min, the upper solution (400 μl) was then evaporated, and the sample was finally redissolved in 100 μl methanol. For the serum analyzed by Hydrop interaction liquid chromatography (HILIC), 50 μl plus 150 μl acetonitrile was added to the centrifuge tube to precipitate the protein, and 100 μl of the upper solution was centrifuged under the above-mentioned conditions to be determined.

### Chromatographic Condition

For the C18 separation, mobile phase A consisted of acetonitrile/water (60/40), and mobile phase B was isopropanol/acetonitrile (90/10); both A and B contained 0.1% formic acid and 10 mmol/L ammonium acetate. The column was an HSS T3 column (2.1 × 100 mm, 1.8 μm, Waters) operated at 45°C. The flow rate was 300 μl/min, and the injection volume was 1 μl. For the HILIC separation, mobile phase A was acetonitrile, and mobile phase B was water; both A and B contained 0.1% formic acid and 10 mmol/L ammonium acetate. The column was a BEH amide column (2.1 × 100 mm, 1.7 μm, Waters) operated at 40°C. The flow rate was 300 μl/min, and the injection volume was 1 μl.

### LC–MS Detection

A metabolomics analysis was performed using a Thermo Scientific Q Exactive hybrid quadrupole Orbitrap mass spectrometer equipped with a HESI-II probe. The positive and negative HESI-II spray voltages were 3.7 and 3.5 kV, respectively, the heated capillary temperature was 320°C, the sheath gas pressure was 30 psi, the auxiliary gas setting was 10 psi, and the heated vaporizer temperature was 300°C. Both the sheath gas and the auxiliary gas consisted of nitrogen. The collision gas was also nitrogen at a pressure of 1.5 mTorr. The parameters of the full mass scan were as follows: resolution of 70,000, auto gain control target under 1 × 106, maximum isolation time of 50 ms, and m/z range of 50–1,500. The LC–MS system was controlled using Xcalibur 2.2 SP1.48 software (Thermo Fisher Scientific), and data were collected and processed using the same software.

### Untargeted Metabolome Data Processing

All data obtained from the four assays in the two systems in both positive and negative ion modes were processed using Progenesis QI data analysis software (Non-linear Dynamics, Newcastle, United Kingdom) to impute raw data, peak alignment, picking, and normalization to produce peak intensities for retention time (*t*_*R*_) and m/z data pairs. The ranges of automatic peak picking for the C18 were between 1 and 16 min and between 1 and 12 min, respectively. Next, the adduct ions of each “feature” (m/z, *t*_*R*_) were deconvoluted, and these features were identified in the human metabolome database (HMDB) and Lipidmaps.

To monitor a system’s stability and performance and the reproducibility of the sample, quality control (QC) samples were prepared by pooling equal volumes of each serum sample. The pretreatment of serum QC samples was performed in accordance with real samples. For repeatable metabolic analyses, three features of the analytical system must be stable: (1) retention time, (2) signal intensity, and (3) mass accuracy. In this study, three QCs were continuously injected at the beginning of the run. QC samples are then injected at regular intervals of six or eight samples throughout the analytical run to provide data from which repeatability can be assessed.

The features were selected based on their coefficients of variation (CVs) with QC samples; features with CVs over 15% were eliminated.

### Statistical Analysis

The classified variables and continuous variables in the baseline information on participants were compared by χ^2^ test and *t*-test in SPSS, respectively. Data are presented as mean ± SD or the percentage, as appropriate. A multivariate statistical analysis was performed using principal component analysis (PCA) and orthogonal projections to latent structures—discriminant analysis (OPLS-DA) multivariate statistical methods in SIMCA (14.1) software. In this study, the variable importance in the projection (VIP) value of the OPLS-DA model (threshold > 1) and the *P-*value of *t*-test (*P* < 0.05) were used to identify the different metabolites. The qualitative method of different metabolites consists of searching in HMDB (to compare the m/z or molecular mass, error limit 0.01 Da). The OPLS-DA model was then validated by permutation tests. A pathway analysis was performed using MetaboAnalyst 4.0.

## Results

### Baseline Characteristics

In this study, there were 99 people in the AIS group and 50 people in the control group. The baseline characteristics are shown in Table 1. There were 73 males and 26 females with an average age of 58.06 years in the AIS group and 36 men and 14 women with an average age of 57.60 years in the control group. We found no significant difference in age, sex, hyperlipidemia, and diabetes mellitus between the AIS and control groups (*P* > 0.05).

**TABLE 1:** Comparison of baseline characteristics between the patients and the healthy controls.

		**Patients (*n* = 99)**		**Controls (*n* = 50)**	***P-*value**
		
	**LAA (*n* = 49)**	**SAO (*n* = 50)**	**Total**		
Age (years), mean ± SD	58.08 ± 12.827	58.04 ± 10.234	58.06 ± 11.531	57.60 ± 2.718	0.781
Male, *n* (%)	36 (73.5)	37 (74.0)	73 (73.7)	36 (72.0)	0.821
**Medical history, *n* (%)**					
Hypertension	29 (52.9)	24 (48.0)	53 (53.5)	13 (26.0)	0.001
Diabetes	13 (26.5)	7 (14.0)	20 (20.2)	8 (16.0)	0.535
Hyperlipidemia	5 (10.2)	4 (8.0)	9 (9.1)	8 (16.0)	0.210
Coronary heart disease	3 (6.1)	2 (4.0)	5 (5.1)	1 (2.0)	0.664
Smoking	21 (42.9)	20 (40.0)	41 (41.4)	9 (18.0)	0.004
Drinking	18 (36.7)	19 (38.0)	37 (37.4)	16 (32.0)	0.518

*LAA, large artery atherosclerosis; SAO, small artery occlusion.*

### PCA and OPLS-DA

**FIGURE 1:**
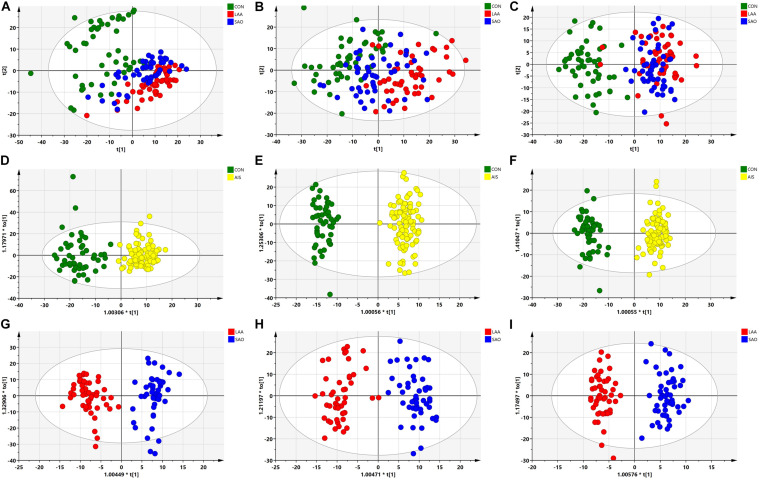
Multivariate statistical analysis of serum metabolic profiling between acute ischemic stroke (AIS) and healthy control (HC) groups. **(A)** Principal component analysis (PCA) score plots in the C18-positive column (*R*^2^*X* = 0.789, *Q*^2^ = 0.547). **(B)** PCA score plots in the C18-negative column (*R*^2^*X* = 0.817, *Q*^2^ = 0.616). **(C)** PCA score plots in the Hydrop interaction liquid chromatography (HILIC) column (*R*^2^*X* = 0.732, *Q*^2^ = 0.453). **(D)** Orthogonal projections to latent structures—discriminant analysis (OPLS-DA) score plots of patients with AIS and HCs in the C18-positive column (*R*^2^*Y* = 0.883, *Q*^2^ = 0.726). **(E)** OPLS-DA score plots of patients with AIS and HCs in the C18-negative column (*R^2^Y* = 0.964, *Q*^2^ = 0.857). **(F)** OPLS-DA score plots of the patients with AIS and HCs in the HILIC column (*R*^2^*Y* = 0.985, *Q*^2^ = 0.914). **(G)** OPLS-DA score plots of the large artery atherosclerosis (LAA) and the small artery occlusion (SAO) groups in the C18-positive column (*R*^2^*Y* = 0.916, *Q*^2^ = 0.778). **(H)** OPLS-DA score plots of the LAA and the SAO groups in the C18-negative column (*R*^2^*Y* = 0.909, *Q*^2^ = 0.800). **(I)** OPLS-DA score plots of the LAA and the SAO groups in the HILIC column (*R*^2^*Y* = 0.953, *Q*^2^ = 0.726).

The PCA of the unsupervised model was used to analyze the differences and intra-group variation among the LAA, SAO, and HC groups, in which *R*^2^*X* was used to judge the quality of the model, and *Q*^2^ represented the predictable variables of the model. As shown in Figures 1A–C, there was a slight separation among the three groups on the score plots in both the C18 column and the HILIC column (C18-positive: *R*^2^*X* = 0.789, *Q*^2^ = 0.547; C18-negative: *R*^2^*X* = 0.817, *Q*^2^ = 0.616; HILIC: *R*^2^*X* = 0.732, *Q*^2^ = 0.453). To obtain the metabolite information that leads to this difference, supervised models and OPLS-DA were performed. The serum samples in the AIS group and the HC group were separated in the C18 column and HILIC column (C18-positive: *R*^2^*Y* = 0.883, *Q*^2^ = 0.726; C18-negative: *R*^2^*Y* = 0.964, *Q*^2^ = 0.857; HILIC: *R*^2^*Y* = 0.985, *Q*^2^ = 0.914) as were the LAA group and the SAO group (C18-positive: *R*^2^*Y* = 0.916, *Q*^2^ = 0.778; C18-negative: *R*^2^*Y* = 0.909, *Q*^2^ = 0.800; HILIC: *R*^2^*Y* = 0.953, *Q*^2^ = 0.726), highlighting the excellence of the models (Figures 1D–I).

### Different Metabolites

The analysis of OPLS-DA in the supervised model is summarized in Table 2. A total of 18 significantly changed metabolites (SCMs) (VIP > 1, *P* < 0.05) were screened between the AIS group and the HC group through different chromatographic columns, and a total of eight SCMs were screened between the LAA and SAO groups from the HMDB.

**TABLE 2:** Characteristics of the different metabolites.

**Metabolites**	**Retention time (min)**	**Mass-to-charge ratio**	**VIP value**	**Fold change**	***P*-value**
**Between the AIS and HCs groups**					
Oleic acid	5.116	281.249	2.107	2.216	<0.00001
Linoleic acid	4.465	279.233	1.844	1.970	0.00001
Cer (d18:0/14:0)	8.993	512.503	1.735	0.212	<0.00001
Cer (d18:0/16:0)	9.546	540.534	1.732	0.213	<0.00001
Arachidonic acid	4.421	303.233	1.556	1.564	0.00030
Non-adecanoic acid	6.306	297.280	1.371	0.652	<0.00001
Docosahexaenoic acid	4.207	327.233	1.306	1.342	0.00964
4-Hydroxyproline	5.939	132.066	1.270	0.520	<0.00001
PE[18:2(9Z,12Z)/18:1(9Z)]	8.534	742.538	1.099	0.492	<0.00001
PE[18:2(9Z,12Z)/18:0]	8.989	744.553	1.099	0.514	<0.00001
L-Palmitoylcarnitine	1.433	400.342	1.098	1.338	<0.00001
Propionylcarnitine	2.604	218.139	1.097	0.716	<0.00001
Tetradecanoylcarnitine	1.473	372.311	1.095	1.382	0.00015
L-Glutamine	6.353	147.077	1.094	0.810	<0.00001
L-Arginine	7.316	175.119	1.093	0.742	<0.00001
Dodecanoylcarnitine	1.522	344.280	1.094	1.349	0.00695
L-Proline	5.346	116.071	1.092	0.758	<0.00001
Decanoylcarnitine	1.593	316.248	1.090	1.330	0.02110
**Between the LAA and SAO group**					
L-Pipecolic acid	4.735	130.087	1.578	1.790	0.00065
1-Methylhistidine	7.267	170.093	1.317	1.625	0.04886
PE [22:6(4Z,7Z,10Z,13Z,16Z,19Z)/16:0]	8.209	764.521	1.270	0.780	0.02704
PE [P-18:0/18:2(9Z,12Z)]	9.314	728.557	1.192	1.276	0.01645
LysoPE [18:2(9Z,12Z)/0:0]	2.428	478.292	1.113	1.446	0.00011
LysoPC [18:3(9Z,12Z,15Z)]	1.991	518.323	1.045	1.387	0.00144
LysoPC (20:0)	5.009	552.402	1.033	1.254	0.00669
LysoPC [18:2(9Z,12Z)]	2.455	520.339	1.000	1.336	0.00013

*LAA, large artery atherosclerosis; SAO, small artery occlusion; AIC, acute ischemic stroke; HC, healthy control; PE, phosphatidylethanolamine.*

**FIGURE 2:**
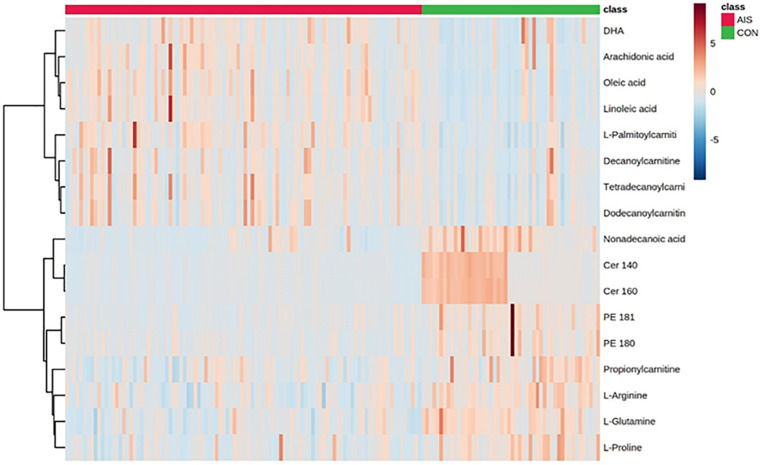
Heat map of different serum metabolites between patients with acute ischemic stroke and healthy control groups.

Compared with the HCs, the AIS patients exhibited higher levels of oleic acid, linoleic acid, arachidonic acid (AA), docosahexaenoic acid (DHA), L-palmitoylcarnitine, tetradecanoylcarnitine, dodecanoylcarnitine, and decanoylcarnitine and lower levels of Cer (14:0), Cer (16:0), non-adecanoic acid, 4-hydroxyproline, phosphatidylethanolamine (PE) (18:1), PE (18:0), propionylcarnitine, L-glutamine, L-arginine, and L-proline as shown in the heat map in Figure 2. In comparison to the SAO group, the LAA group was characterized by decreased levels of L-pipecolic acid, 1-methylhistidine, PE (18:2), LysoPE (18:2), LysoPC (18:3), LysoPC (20:0), and LysoPC (18:2) and by increased levels of PE (16:0). Detailed information is shown in Table 2.

### Metabolic Pathways

**FIGURE 3:**
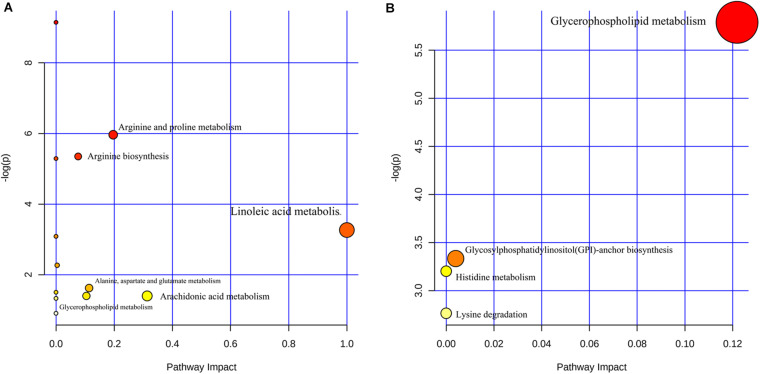
Different metabolic pathways **(A)** between patients with acute ischemic stroke and healthy control groups and **(B)** between the large artery atherosclerosis and the small artery occlusion groups.

MetaboAnalyst 4.0 was used to analyze the different metabolic pathways of the groups. The potential different metabolic pathways between the AIS patients and the HCs include linoleic acid metabolism, AA metabolism, arginine and proline metabolism, and alanine, aspartate, and glutamate metabolism. The metabolic pathways of the LAA group and the SAO group probably differ in glycerophospholipid metabolism, glycosylphosphatidylinositol (GPI)-anchor biosynthesis, histidine metabolism, and lysine degradation (Figure 3).

## Discussion

We obtained the serum metabolic profiling of stroke patients by non-targeted metabolomics and discovered that the AIS patients had metabolic disorders. Furthermore, the metabolic profiles of the LAA and SAO subtypes of IS were different. Among the abnormal metabolic indicators, the metabolic disorders of lipids and amino acids are the most obvious. Changes in metabolite patterns lay the foundation for us to further clarify the pathophysiological mechanisms of stroke and find ways to implement innovative clinical diagnoses of stroke classifications.

The brain consumes approximately 20% of total human energy consumed. Approximately 20% of the energy consumed by the brain is provided by the oxidative reaction of fatty acids (Ebert et al., 2003). Neurons are very sensitive to conditions such as ischemia and hypoxia. In order to regulate the lack of energy caused by the AIS, the brain can initiate energy production responses such as fatty acid degradation through negative feedback to maintain homeostasis (Schwartz et al., 2000; Belgardt and Brüning, 2010). Oleic acid and linoleic acid are long-chain fatty acids that can cross the blood–brain barrier to provide energy to the brain (Panov et al., 2014), and L-palmitoylcarnitine is also involved in fatty acid degradation. In this study, compared with the HC group, the increase in oleic acid, linoleic acid, and L-palmitoylcarnitine in the AIS group may be associated with increased fatty acid catabolism in the acute phase of the IS to maintain energy homeostasis. In addition, previous studies have reported that changes in lipid metabolism are associated with mitochondrial dysfunction caused by oxidative stress (Tobe, 2013), which is one of the three main pathophysiological reactions (neurotoxicity, oxidative stress, and inflammation) in IS (Fukuyama et al., 1998; Chamorro et al., 2012; Lai et al., 2014). This result is consistent with the transcriptomic profiling results of IS (Li et al., 2015; Cai et al., 2019). Cai et al. (2019) assessed the patterns of transcriptomic changes at different stages of IS using a mouse model. The results showed that mmu-miR-199a-5p and mmu-miR-199b-3p inhibit the inflammatory response during the recovery phase of IS and exert neuroprotective effects by regulating the Taok1 gene (Cai et al., 2019). This may imply that fatty acid metabolism is related to the regulation of these genes. However, this requires further verification in animal experiments.

Both AA and DHA are polyunsaturated fatty acids which are released from the metabolic pathway of glycerol phospholipid degradation and are the main components of the phospholipid membrane. AA and DHA participate in membrane fluidity, signal transduction, and gene transcription during the whole life process (Rapoport et al., 2001). They are also involved in many pathological processes, including stroke (Rapoport, 2008). AA is stored in the phospholipid membranes of cells, which produce free AA by deacylation mediated by phospholipase A2 (PLA2). In pathological environments such as stroke, free AA increases the production of free radicals through the “arachidonic acid cascade reaction” (Rink and Khanna, 2011). This reaction can occur as early as 1 h after a stroke (Shohami et al., 1982), a major factor in the oxidative damage of tissues after a stroke. Consistent with the results of our study, previous studies reported a significant increase in the types of reactive oxygen species and AA metabolism after reperfusion in IS (Gürsoy-Ozdemir et al., 2004).

Ceramide is a type of waxy lipid composed of sphingosine and fatty acids, which plays a role in plaque formation (Holland and Summers, 2008). In addition, ceramide levels in the high-risk groups with IS were higher than those in the low-risk groups (Wang et al., 2017). However, previous studies reported that the level of ceramide in patients with AIS is lower than that in the HC group, which may be related to ceramide-mediated apoptosis (Taha et al., 2006). The specific mechanism warrants further study. Glutamine (Gln) and glutamate (Glu) can be converted into each other in the human body. The level of glutamine and the ratio of Gln to Glu were negatively correlated with the risk factors of cardiovascular disease (including body mass index, waist circumference, fasting blood glucose, insulin, triglyceride, *etc*.) (Cheng et al., 2012). Zheng et al. (2016) demonstrated that the ratio of Gln to Glu was associated with a reduced risk of cardiovascular disease. Similarly, glutamine levels in the AIS group were lower than those in the HC group of our study.

In the different metabolites of the LAA group and the SAO group, L-pipecolic acid mainly affects the lysine degradation pathway because lysine is produced in the process of L-pipecolic acid degradation, and lysine has previously been shown to decrease in patients with IS (Kimberly et al., 2013; Lee et al., 2017). 1-Methylhistidine is involved in histidine metabolism, which is a metabolic byproduct of the antioxidant molecule carnosine and its analogs in the brain (Bellia et al., 2011). Hu et al. (2019) showed that the level of L-pipecolic acid in patients with post-stroke depression is lower than that in HCs but higher than that in patients with stroke. PE, LysoPC, and LysoPE participate in glycerophospholipid metabolism and GPI-anchor biosynthesis. LysoPE is a product of PE hydrolyzed by PLA2, which plays a role in cell-mediated cell signaling and the activation of other enzymes (Park et al., 2007). PE and LysoPC are intermediate products of glycerophospholipid metabolism, while AA and DHA are glycerophospholipid degradation products. It should be noted that the metabolic changes of glycerophospholipids can not only help diagnose AIS but also help distinguish different subtypes of IS. This might shed insight on our exploration of the pathological mechanisms of different subtypes. Studies have confirmed the correlation between lipid metabolites and AIS using lipidomic and metabolomics techniques (Yang et al., 2017; Au, 2018). Our results are consistent with these (Liu et al., 2017). To date, there has been no research exploring the mechanisms of different metabolite isomers showing different behaviors. This may require further lipidomic assessments in a larger sample size and further verification in animal models.

In this study, non-targeted metabolomics based on LC–MS was used to identify the different metabolites between patients with AIS and the HCs and between the LAA and SAO groups, providing new insights and encouraging further study of the pathophysiological mechanisms among different subtypes of IS. However, this study still has many limitations. First, this was a single-center study with a relatively small sample size. Multicenter studies with larger sample sizes will be needed to validate our findings. In addition, because the metabolites in the human body change dynamically, we collected the serum after the onset of the disease, which may have affected the estimation of the correlation between the metabolic differences and the disease. A longitudinal comparison of multiple blood samples, after the onset, from the same patient will enable a clearer assessment of the changes in serum metabolites in AIS patients. Finally, targeted metabolomics technology in another set of samples is needed to further verify the different metabolites. In the future, to validate the results and investigate the potential of metabolites as biomarkers, we will include patients and follow them up prospectively to obtain their modified Rankin Scale scores and further explore metabolites associated with prognosis.

## Conclusion

In summary, this study identified the different metabolites and metabolic pathways in patients with AIS and HCs and between the LAA and SAO subtypes of IS by non-targeted metabolomics. We demonstrated that metabolomics might be used to diagnose AIS and distinguish its subtypes. Further research is needed to explore the pathophysiological mechanisms that affect the changes in metabolites and lead to new clinical diagnoses and potential interventions.

## Data Availability Statement

The raw data supporting the conclusions of this article will be made available by the authors, without undue reservation.

## Ethics Statement

The studies involving human participants were reviewed and approved by The First Affiliated Hospital of Zhengzhou University. The patients/participants provided their written informed consent to participate in this study.

## Author Contributions

XW, BS, and J-SK conceived and designed the research. XW, MT, and LZ conducted the experiments. XW, MT, LZ, L-lP, WS, and JL performed the data collection. XW, XL, RZ, JW, and SS analyzed the data. XW wrote the manuscript. All authors have read and approved the manuscript.

## Conflict of Interest

The authors declare that the research was conducted in the absence of any commercial or financial relationships that could be construed as a potential conflict of interest.
